
JOURNAL INFORMATION
==============================
NLM Title Abbreviation: Anim Front
Journal ID: Anim Front
NLM Journal ID: 101754918
Journal ID: af

Animal Frontiers: The Review Magazine of Animal Agriculture

ISSN: 2160-6056
EISSN: 2160-6064
Publisher: Oxford University Press

ARTICLE INFORMATION
==============================
PMCID: PMC6952869
PMID: 32002205
DOI: 10.1093/af/vfz046
Article ID: vfz046
Article version: 1
Subjects: Society News

News from the Canadian Society of Animal Science

Publication date: 2020 Jan
Electronic publication date: 2020 Jan 10
Volume: 10
Issue: 1
First page: 60
Last page: 60
Copyright: Copyright © 2020 American Society of Animal Science
Copyright year: 2020
License: This is an Open Access article distributed under the terms of the Creative Commons Attribution License (http://creativecommons.org/licenses/by/4.0/), which permits unrestricted reuse, distribution, and reproduction in any medium, provided the original work is properly cited.
License URL: https://creativecommons.org/licenses/by/4.0/

Page count: 1

==============================
### News from the Canadian Society of Animal Science

Dear Members and Partners,

The Canadian Journal of Animal Science has been published since 1957. This quarterly journal contains new research on all aspects of animal agriculture and animal products, including breeding and genetics, cellular and molecular biology, growth and development, meat science, modelling animal systems, physiology and endocrinology, ruminant and non-ruminant nutrition, welfare, behaviour, and management.

We encourage our members to submit their original manuscripts to the Canadian Journal of Animal Science!

### Worth a read:

Read Livestock Gentec's interesting article on the development of genomic breeding value estimation here: Happy Birthday, gEBVs!

### Upcoming events:

October 1-5. World Dairy Expo – Madison, WI, USA
October 8. Canadian Agricultural Safety Association Annual Conference. Ottawa, ON
October 19-21. Leman China Swine Conference. Zhengzhou, China
October 23-23. 129th World Food Science & Food Technology Congress. Toronto, ON
October 27-19. Advancing Women in Agriculture Conference – East. Niagara Falls, ON

### Thank you to our sponsors:

doi: 10.1093/af/vfz046
